# TyG index combined with BMI predicts metabolism dysfunction-associated fatty liver disease in patients with type 2 diabetes

**DOI:** 10.1515/med-2026-1457

**Published:** 2026-06-18

**Authors:** Qian Gao, Lei Feng, Weiling Zhou, Xiaoli Li, Lanzi Yin, Xiaojuan Hou, Yuan Wang

**Affiliations:** Department of Endocrine and Metabolic Diseases, The Fourth Hospital of Hebei Medical University, Shijiazhuang, China

**Keywords:** TyG index(triacylglycerol-glucose index), BMI(body mass index), metabolism dysfunction-associated fatty liver disease (MAFLD), type 2 diabetes mellitus (T2DM)

## Abstract

**Objectives:**

To explore the predictive capacity of the TyG index (triacylglycerol-glucose index) in conjunction with BMI for diagnosing MAFLD among patients with type 2 diabetes mellitus (T2DM).

**Methods:**

A retrospective study of 356 consecutive type-2 diabetes patients followed by the Endocrinology Department from May 2022 to May 2024 was studied. Participants were divided into MAFLD and non-MAFLD groups, and the association and predictive accuracy of the TyG index were evaluated using logistic regression and ROC curve analyses.

**Results:**

Significant variations in the incidence of MAFLD were observed across the quartiles Q1 (TyG<8.98), Q2 (8.98≤TyG<9.36), Q3 (9.36≤TyG<9.94), and Q4 (TyG≥9.94) (p<0.001). The formula log (p)=−22.549+0.385 * BMI + 1.3708 * TyG index illustrated that the TyG index and BMI are predictive of the likelihood of MAFLD in patients with T2DM. Furthermore, the TyG index and BMI were strongly correlated with MAFLD, evidenced by an area under the curve of 0.868 (95 % CI [0.829, 0.907], p<0.001), a sensitivity of 83.0 %, and a specificity of 77.8 %.

**Conclusions:**

The TyG index is significantly associated with the risk of developing MAFLD in individuals with type 2 diabetes, and its predictive utility is markedly enhanced when BMI is incorporated.

## Introduction

In recent years, the expansion of international markets and substantial shifts in lifestyle habits have led to an increased prevalence of conditions such as MAFLD (metabolism dysfunction-associated fatty liver disease), Type 2 diabetes mellitus (T2DM), and obesity [1]. MAFLD, formerly referred to as non-alcoholic fatty liver disease (NAFLD), represents the most common chronic liver condition globally, affecting approximately a quarter of the global population [2]. Characterised as a metabolic disorder, MAFLD can progress to liver fibrosis, cancer, and ultimately, death. The renaming of NAFLD to MAFLD underscores its metabolic basis and aids in the development of targeted metabolic treatments [3], [4], [5]. Early detection of MAFLD is crucial for alleviating its economic burden and delaying disease progression. Consequently, the adoption of convenient and non-intrusive diagnostic tools is imperative for early intervention, which in turn, can mitigate the severity of the disease and reduce associated costs. Given the shared pathological pathways between T2DM and MAFLD, including liver glycogen anomalies and insulin resistance (IR), it is essential to identify and predict MAFLD in patients with Type 2 diabetes mellitus [6], 7]. Simental [8] identified the TyG index (triacylglycerol-glucose index) as a viable marker for assessing IR. While a high TyG index has been linked to NAFLD, limited data exists on its relationship with MAFLD, particularly in diabetic patients [9], [10], [11]. Recent studies have explored the use of similar combinations (e.g., TyG index with other metabolic markers) in metabolic disease populations, but few have focused specifically on T2DM patients. Our study aims to fill this gap by investigating the predictive capacity of the TyG index combined with BMI for diagnosing MAFLD in patients with T2DM, thereby providing a practical and effective tool for early detection and intervention. This study demonstrates a significant correlation between the TyG index and MAFLD, suggesting that the TyG index, in conjunction with BMI, can effectively predict the presence of MAFLD. This finding complements existing literature by providing a practical and effective predictive model specifically for T2DM patients. While previous studies have explored similar combinations in metabolic disease populations, our study uniquely focuses on the diabetic population, offering a novel approach for early detection and intervention. Future research should aim to validate this model in diverse cohorts and settings to confirm its robustness and applicability.

## Materials and methods

### Study design and patients

A retrospective study encompassed 356 individuals with T2DM who were evaluated in the Endocrinology Department from May 2022 to May 2024. Among these, 212 were diagnosed with MAFLD, while the remainder exhibited no evidence of the disease. Eligibility criteria included being 18 years of age or older, type 2 diabetes mellitus diagnosed according to the 1999 WHO criteria, and fatty liver confirmed by a certified sonographer in our hospital’s ultrasound department with the Philips EPIQ 7 system. All MAFLD patients were included only when both of the following ultrasound criteria were met in at least two separate planes (right and left liver): (1) increased hepato-renal echo contrast and (2) posterior attenuation. All enrolled MAFLD patients were rigorously screened to exclude other etiologies of hepatic steatosis (e.g., alcohol excess, hepatotoxic medications) and were required to have ≥1 metabolic risk factor (obesity, type 2 diabetes, or dyslipidemia). Exclusion criteria comprised patients with acute coronary syndrome, acute cerebral infarction, cerebral haemorrhage, cancer, severe infections, or significant liver or kidney dysfunction; those with a history of liver surgery; individuals with mental health conditions impeding study compliance; or those with incomplete data collection [12]. Subjects with poor image quality – i.e., liver margins or parenchyma could not be clearly visualized owing to abundant abdominal fat, bowel gas, or other artifacts were excluded. The diagnostic criteria for MAFLD were established using liver biopsy, imaging studies, and blood biomarkers, in conjunction with at least one of the following: overweight/obesity, T2DM, or metabolic dysfunction. These criteria encompassed: 1) a waist circumference of ≥90 cm for Asian men and >80 cm for women, 2) blood pressure of ≥130/85 mmHg or the use of antihypertensive medications, 3) blood triglycerides of ≥1.70 mmol/L or the use of lipid-lowering treatments, 4) plasma HDL-C levels of <1.0 mmol/L for men and <1.3 mmol/L for women, or the use of lipid-regulating medications, 5) prediabetes, defined as a fasting blood glucose (FBG) level of 5.6–6.9 mmol/L, a 2-hour postprandial blood glucose level of 7.8–11.0 mmol/L, or an HbA1c level of 5.7–6.4 %, 6) an islet resistance index of ≥2.5 as determined by the steady-state model, and 7) hypersensitive C-reactive protein (hsCRP) levels of >2 mg/L [5]. In individuals who are not overweight or obese, the presence of two or more risk factors for metabolic abnormalities, in conjunction with fatty liver disease and a normal BMI, also qualifies as diagnostic criteria for MAFLD.

The study adhered to the principles outlined in the Declaration of Helsinki and received approval from the Ethical Committee of The Fourth Hospital of Hebei Medical University (2022KY402). Due to the retrospective nature of the study, the requirement for informed consent was waived.

### Data collection and definitions

Data collected on the study participants included age, gender, duration of T2DM, history of hypertension, use of lipid-regulating and liver protection medications, as well as measurements of height, weight, and blood pressure. Fasting venous blood samples were obtained for analysis of global measure of circulating cholesterol including HbA1c, total cholesterol (TC), triglycerides (TG), low-density lipoprotein cholesterol (LDL-C), high-density lipoprotein cholesterol (HDL-C); traditional hepatic injury markers including alanine aminotransferase (ALT), alkaline phosphatase (ALP), aspartate aminotransferase (AST), homocysteine (HCY); sugar metabolism. (fasting blood glucose, FBG); lipid metabolism lipoprotein alpha, and kidney function, Creatinine (Cr), and blood uric acid (BUA) [12], 13]. The TyG index was calculated using the formula: TyG=Ln [(TG (mg/dL) × FBG (mg/dL))/2]; BMI was determined by the formula: weight (kg)/height^2^ (m^2^) [8].

We Summarize of patient clinical profile and key indicators in Table 1. Based on the TyG quartiles, patients were categorised into Q1 (TyG<8.98), Q2 (8.98≤TyG<9.36), Q3 (9.36≤TyG<9.94), and Q4 (TyG≥9.94). Comparison of MAFLD Incidence Across Different TyG Index Categories (Table 2.) We analyzed the correlation between triglyceride-glucose (TyG) index and other clinical parameters in Table 3. Binary logistic regression was used to determine the factors related to MAFLD in type 2 diabetes patients (Table 4).

**Table 1:** Summary of patient clinical profile and key indicators.

Index	Totality (n=356)	MAFLD (n=212)	Non-MAFLD (n=144)	X^2^/z	p-Value
Gender				13.190	<0.001^a^
Male	237 (66.6 %)	157 (74.1 %)	80 (55.6 %)		
Female	119 (33.4 %)	55 (25.9 %)	64 (44.4 %)		
Hypertension	294 (82.6 %)	163 (76.9 %)	101 (70.1 %)	2.037	0.153
The duration of diabetes				10.530	0.005^a^
<5 years	174 (48.9 %)	98 (46.2 %)	76 (52.8 %)		
5–10 years	102 (28.7 %)	54 (25.5 %)	48 (33.3 %)		
≥10 years	80 (22.5 %)	60 (28.3 %)	20 (13.9 %)		
Age	52 (43, 61)	51 (39.25, 61)	54.5 (43, 60)	−1.915	0.056
BMI, kg/m^2^	26.33 (23.78, 29.74)	28.07 (25.92, 30.41)	23.78 (21.63, 25)	−10.407	0.000^a^
SBP, mmHg	138 (121, 150)	142 (120, 149)	141.5 (124.25, 150)	−1.343	0.219
DBP, mmHg	83.5 (75, 89)	82 (73.25, 88)	85 (77, 90)	−1.095	0.236
TC, mmol/L	4.89 (4.02, 5.87)	4.9 (4.15, 5.99)	4.6 (3.37, 5.78)	−2.679	0.007^a^
LDL, mmol/L	3.17 (2.52, 3.98)	3.25 (2.7, 3.98)	2.85 (2.11, 3.92)	−2.182	0.029^a^
HDL, mmol/L	1.1 (0.9, 1.3)	1.11 (0.85, 1.35)	1.09 (0.9, 1.3)	−0.639	0.523
TG, mmol/L	1.89 (1.27, 2.79)	2.5 (1.56, 3.29)	1.4 (1.01, 1.96)	−7.924	0.000^a^
ALT, U/L	26.1 (19.5, 43.4)	25.1 (19.3, 40.7)	28.15 (20.35, 43.4)	−0.467	0.641
AST, U/L	19.4 (15.3, 25.8)	19.3 (15.6, 24.9)	19.5 (14.25, 25.8)	−0.758	0.449
HCY, umol/L	14.2 (11.3, 16.7)	14.1 (11.2, 16.7)	14.4 (12.1, 16.7)	−0.120	0.904
FBG, mmol/L	8.31 (6.47, 10.15)	8.89 (6.66, 11.75)	7.76 (5.89, 9.04)	−3.986	0.000^a^
HbA1c %	8.3 (6.8, 10.8)	8.6 (6.9, 11.1)	7.6 (6.3, 9.7)	−4.018	0.000^a^
Cr, umol/L	61.3 (49.7, 68.5)	62 (51.3, 70.3)	60.3 (48.73, 66.1)	−1.899	0.058
ALP, U/L	78 (60.2, 98.6)	82.4 (67.2, 97.7)	77.9 (56.6, 98.6)	−0.483	0.629
Lip-α, mg/L	159.8 (103.6, 310.4)	159.8 (109.35, 310.4)	159.8 (91.2, 266.9)	−1.040	0.298
BUA, umol/L	335.7 (276.1, 417.6)	341.6 (297.5, 428.3)	301.5 (240.98, 385.8)	−3.871	0.000^a^
TyG	9.36 (8.98, 9.94)	9.68 (9.16, 10.2)	8.99 (8.64, 9.44)	−8.332	0.000^a^

^a^P value less than 0.05 was considered statistically significant; Quantitative variables are shown as median (interquartile range), and qualitative parameters are presented as numbers with the percentage in parentheses. SBP, systolic blood pressure; DBP, diastolic blood pressure; BMI, body mass index; FBG, fasting plasma glucose; HbA1c, glycated hemoglobin; TG, total triglyceride; TC, total cholesterol; HDL-C, high density lipoprotein-cholesterol; HCY, homocysteine; ALP, alkaline phosphatase; ALT, alanine aminotransferase; Cr, creatinine; AST, aspartate aminotransferase; LDL-C, low-density lipoprotein-cholesterol; Lip-α,Lipoproteins-α; BUA, blood uric acid.

**Table 2:** Comparison of MAFLD incidence across different TyG index categories.

Group	n	Incidence rate of MAFLD n (%)	X^2^/z	p-Value
Q1	88	27 (30.7)	67.277	<0.001^a^
Q2	79	37 (46.8)		
Q3	103	74 (71.8)		
Q4	86	74 (86.0)		

^a^P value less than 0.05 was considered statistically significant; Qualitative parameters are presented as numbers with the percentage in parentheses.

**Table 3:** Correlation analysis between the triglyceride–glucose (TyG) index and other clinical parameters.

Index	(n=356)		MAFLD (n=212)		Non-MAFLD (n=144)	
	r	p-Value	r	p-Value	r	p-Value
Age	−0.234	0.000^a^	−0.298	0.000^a^	−0.179	0.032^a^
BMI, kg/m2	0.284	0.000^a^	0.122	0.076	0.016	0.848
SBP, mmHg	−0.143	0.057	−0.220	0.071	0.006	0.943
DBP, mmHg	−0.116	0.059	−0.156	0.063	−0.009	0.914
TC, mmol/L	0.447	0.000^a^	0.326	0.000^a^	0.562	0.000^a^
LDL, mmol/L	0.393	0.000^a^	0.252	0.000^a^	0.569	0.000^a^
HDL, mmol/L	0.163	0.072	0.229	0.061	0.152	0.070
TG, mmol/L	0.827	0.000^a^	0.749	0.000^a^	0.790	0.000^a^
ALT, U/L	0.073	0.169	0.195	0.004^a^	−0.015	0.862
AST, U/L	0.143	0.007^a^	0.147	0.032^a^	0.200	0.016^a^
HCY, umol/L	0.021	0.689	0.077	0.263	0.014	0.864
Cr, umol/L	−0.065	0.221	−0.031	0.652	−0.260	0.002^a^
ALP, U/L	0.055	0.298	−0.011	0.868	0.128	0.127
Lip-α, mg/L	−0.084	0.113	−0.105	0.128	−0.195	0.019^a^
BUA, umol/L	0.148	0.005^a^	0.103	0.134	0.026	0.757

^a^P value less than 0.05 was considered statistically significant. SBP, systolic blood pressure; DBP, diastolic blood pressure; BMI, body mass index; FBG, fasting plasma glucose; HbA1c, glycated hemoglobin; TG, total triglyceride; TC, total cholesterol; HDL-C, high density lipoprotein-cholesterol; LDL-C, low-density lipoprotein-cholesterol; ALP, alkaline phosphatase; ALT, alanine aminotransferase; Cr, creatinine; Lip-α, Lipoproteins-α; AST, aspartate aminotransferase; HCY, homocysteine; BUA, blood uric acid.

**Table 4:** Results of binary logistic regression analysis identifying factors associated with MAFLD in patients with type 2 diabetes Mellitus.

	B	p-Value	OR (95 %CI)
Hypertension	−0.296	0.59	0.744 (0.253, 2.185)
The duration of diabetes	0.264	0.175	1.302 (0.889, 1.905)
TC, mmol/L	0.208	0.451	1.231 (0.717, 2.112)
LDL, mmol/L	−0.455	0.193	0.635 (0.320, 1.259)
FBG, mmol/L	−0.044	0.613	0.957 (0.807, 1.135)
HbA1c %	0.068	0.350	1.070 (0.928, 1.233)
BMI	0.390	0.000^a^	1.477 (1.328, 1.642)
TG, mmolL	0.542	0.025^a^	1.721 (1.071, 2.762)
BUA, umolL	0.004	0.014^a^	1.004 (1.001, 1.007)
TyG	2.586	0.000^a^	13.272 (3.351, 52.572)

^a^P value less than 0.05 was considered statistically significant. TG, total triglyceride; BUA, blood uric acid; BMI, body mass index; FBG, fasting plasma glucose; HbA1c, glycated hemoglobin; TC, total cholesterol; LDL-C, low-density lipoprotein-cholesterol.

Statistical Analysis: For the statistical analysis, SPSS version 26.0 (IBM Corp., Armonk, NY, USA) was employed. The quantitative data, not following a normal distribution, were presented as median (P25, p75), while categorical data were denoted as n (%). The Mann-Whitney U test facilitated the comparison between two groups. The chi-square (X^2^) test was utilised for intergroup comparisons, with a significance level set at p<0.05. A logistic regression model identified factors contributing to MAFLD in T2DM patients. The predictive value of the TyG index and BMI for MAFLD was assessed using ROC curve analysis and a joint prediction model [13]. Briefly, factors found to be significantly associated with MAFLD in the univariate analysis were entered into the binary logistic regression model, with variable assignments shown in Table. The ROC curve were calculated between the TyG index and other variables by using SPSS. Binary logistic regression was used to evaluate the association between the TyG index and other variables with MAFLD.

**Table:** 

Variable		Assignment
Dependent variable	MAFLD presence	Yes=1,No=0
Independent variables	Gender	Male=1, Female=2
	Duration of diabetes	Continuous variable
	BMI	Continuous variable
	Total cholesterol (mmol/L)	Continuous variable
	LDL-cholesterol (mmol/L)	Continuous variable
	Triglycerides (mmol/L)	Continuous variable
	Fasting plasma glucose (mmol/L)	Continuous variable
	HbA1c	Continuous variable
	Serum uric acid (µmol/L)	Continuous variable
	TyG index	Continuous variable

### Research ethics

The study was conducted in compliance with the principles outlined in the Declaration of Helsinki and received approval from the Ethical Committee of The Fourth Hospital of Hebei Medical University (2022KY402).

### Informed consent

Not applicable.

## Results

Participant Characteristics: The study included 356 T2DM patients, of which 212 were diagnosed with MAFLD, resulting in a prevalence rate of 59.5 %. Significant differences were observed in the duration of T2DM, TG, FBG, TC, LDL-C, HbA1c, BUA, and TyG index between groups (p<0.05). The MAFLD group consisted of 74.1 % males (157 individuals) and 25.9 % females (55 individuals), compared to 55.6 % males (80 individuals) and 44.4 % females (64 individuals) in the non-MAFLD group, indicating a statistically significant gender distribution (p<0.05). The MAFLD group exhibited significantly higher values for BMI, TG, FBG, TC, LDL-C, HbA1c, BUA, and TyG index (p<0.05) (Table 1).

Relevance Analysis: The prevalence of MAFLD varied significantly across TyG index quartiles, with 30.7 % in Q1, 46.8 % in Q2, 71.8 % in Q3, and 86 % in Q4 (p<0.05) (Table 2). The TyG index was significantly correlated with BMI, TC, LDL-C, AST, HbA1c, and BUA (p<0.05) (Table 3). Factors independently influencing MAFLD development included BMI, TC, BUA, and TyG index (OR>1 and p<0.05) (Table 4).

**Figure 1:**
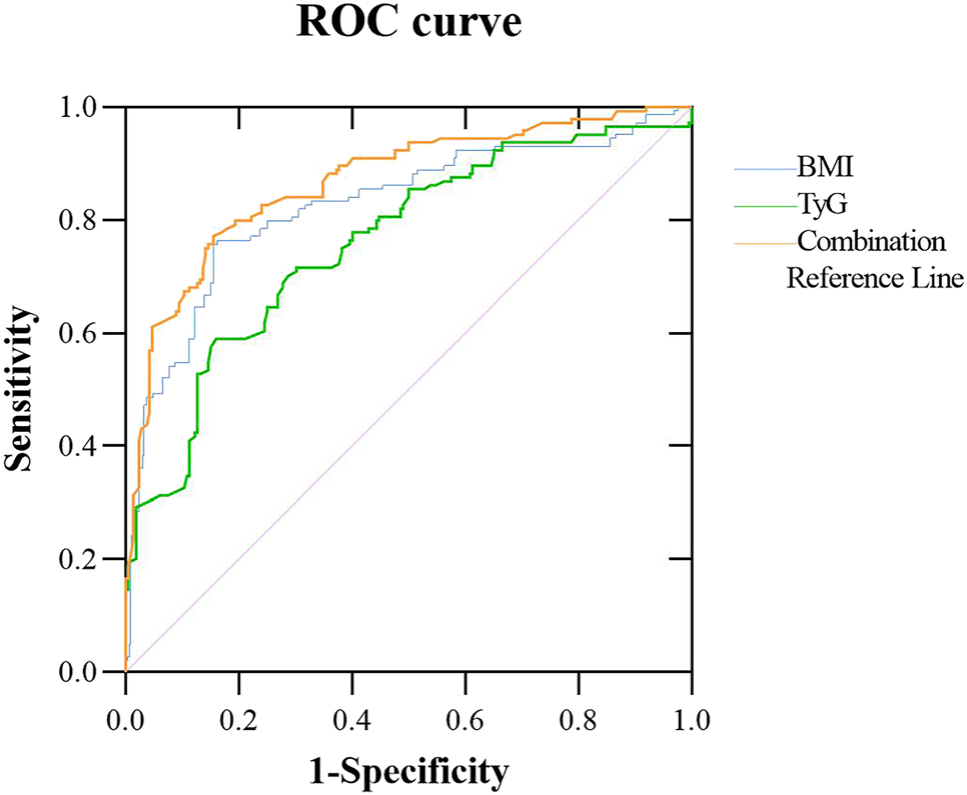
Predictive ROC curves of the TyG index plus BMI for the MAFLD occurrence in patients with T2DM.

Predictive Value: The ROC curve analysis revealed that the TyG index had an AUC of 0.760 (95 % CI: 0.709–0.812, p<0.001) for predicting MAFLD in T2DM patients, with an optimal cutoff value of 9.09, 84.0 % sensitivity, and 59.0 % specificity. Binary logistic regression was used to identify factors associated with NAFLD in patients with T2DM, and receiver operating characteristic (ROC) curve analysis was employed to evaluate the predictive value of BMI combined with TyG index for NAFLD development in this population. When combining the TyG index with BMI, the fitted equation log(p) = −22.549 + 0.385BMI + 1.3708TyG index, and the joint predictor=BMI + (1.3708*TyG index)/0.385. The AUC for the combined prediction was 0.868 (95 % CI: 0.829–0.907, p<0.001), with an optimal cutoff value of 61.77, sensitivity of 83.0 %, and specificity of 77.8 % (Figure 1).

## Discussion

The TyG index has been identified as a reliable marker for assessing insulin resistance (IR), particularly appealing because it obviates the need for insulin quantification and is more practical for individuals undergoing oral medication or insulin therapy [14], 15]. However, the majority of these investigations have focused on non-diabetic and overweight/obese populations, with insufficient depth into the diabetic demographic [16], [17], [18]. Further research is imperative to definitively establish and standardise the TyG index cutoff point [19]. BMI serves as a metric to gauge obesity levels and is correlated with the propensity for metabolic diseases, including insulin resistance, NAFLD, and other associated conditions [20]. In response, we embarked on an investigation to elucidate the relationship between the TyG index and MAFLD and to assess the predictive efficacy of combining TyG and BMI for determining the likelihood of MAFLD in individuals with Type 2 diabetes.

Our study found that 59.5 % of individuals with T2DM were afflicted with MAFLD, consistent with the prevalence rates reported in international literature [6]. The analysis indicated that T2DM patients with elevated TyG indices were more likely to develop MAFLD (p<0.05). Regression analysis revealed that BMI, TC, BUA, and the TyG index were all significantly associated with an increased risk of MAFLD (p<0.05). It is important to consider that the progression and complications associated with diabetes might influence triglyceride and glucose levels. Hence, potential confounders such as the duration of diabetes, history of hypertension, and other pertinent factors were accounted for in the multifactorial analysis. Our findings contribute to the existing body of knowledge by formulating a predictive equation based on a combined predictor for MAFLD onset, derived from transforming the logistic model equation. The TyG-BMI predictor exhibited an area under the curve (AUC) of 0.868, demonstrating a sensitivity of 83.0 % and a specificity of 77.8 %. The integration of TyG-BMI as a predictive measure could significantly improve the precision in identifying MAFLD risk in T2DM patients. This index, easily determinable through routine laboratory tests and measurements, is pragmatically applicable in clinical settings for MAFLD detection. Several studies also found the relationship between TyG index and MAFLD or insulin resistance. Wang et, al. showed that the TyG index could be used in the recognition and diagnosis of metabolism-associated fatty liver disease (MAFLD) [21], with the ability to predict the development of liver fibrosis in affected populations and a high TyG index may contribute to the progression of liver fibrosis [22]. Gao et, al. reported that the TyG-related indexes exhibited the most robust predictive performance among unmarried individuals aged [23]. Our research was in accordance with these results. More detailed clinical insights and enhance the predictive model’s utility for targeted interventions. We plan to explore this in our future studies.

There are several limitations to this study that warrant consideration: 1) The diagnosis of MAFLD via abdominal ultrasound may be subject to variability based on the local adipose tissue content; 2) The study’s cross-sectional design precludes the establishment of causal relationships. The initial levels of triglycerides and glucose could have changed by the time of follow-up, casting uncertainty on whether these changes might yield different outcomes; 3) The investigation did not differentiate between the various stages of fatty liver disease. 4) More external or independent validation would be performed to confirm our results. 5) Although TyG index is a significant predictor of MAFLD, other factors (such as TC, LDL-C, AST, HbA1c, and BUA) might also play a role in predicting MAFLD. For example, some studies found that MAFLD severity is associated with the ratios of total cholesterol and triglycerides [24]. We would conduct more further studies to disentangle the individual contributions of these factors. 6) The lack of external or independent validation was also a limitation. We will conduct future prospective studies with larger sample sizes to further validate these findings. Despite these limitations, this study holds significant clinical importance. A combined predictive model was developed, demonstrating that TyG-BMI is a reliable method for detecting MAFLD in individuals with T2DM. And the TyG-BMI model might serve as an initial “rule-in” and “rule-out” tool, whereas potentially reducing unnecessary referrals and misdiagnosis.

## Conclusions

In summary, this research has established a correlation between the TyG index and the likelihood of MAFLD in patients with Type 2 diabetes. It was indicated that the combination of TyG and BMI offers an accurate predictive tool for the presence of MAFLD.
